# Successful treatment of gemcitabine-induced acute interstitial pneumonia with imatinib mesylate: a case report

**DOI:** 10.1186/s12885-016-2833-9

**Published:** 2016-10-12

**Authors:** Elisabetta Fenocchio, Ilaria Depetris, Delia Campanella, Lucia Garetto, Fabrizio Carnevale Schianca, Danilo Galizia, Giovanni Grignani, Massimo Aglietta, Francesco Leone

**Affiliations:** 1 Department of Medical Oncology, University of Turin Medical School, Candiolo Cancer Institute, FPO, IRCCS, Str. Prov.le 142 Km 3.95, 10060 Candiolo, Turin Italy; 2 Radiology Department, Candiolo Cancer Institute, FPO, IRCCS, Candiolo, Italy

**Keywords:** Gemcitabine, Pancreatic cancer, Pulmonary toxicity, Imatinib mesylate, Treatment outcome

## Abstract

**Background:**

Gemcitabine is currently the standard chemotherapy for the adjuvant treatment of pancreatic cancer. This chemotherapeutic agent is generally well-tolerated, myelosuppression and gastrointestinal toxicity being common side effects. Nevertheless, gemcitabine-induced pulmonary toxicity has been rarely reported. Despite its low incidence, the spectrum of pulmonary injury is wide, including potentially fatal conditions.

We report a case of acute interstitial pneumonia related to gemcitabine, completely solved with Imatinib Mesylate (IM).

**Case presentation:**

The patient was a 69-year-old man, who developed a hypoxemic respiratory distress during adjuvant treatment with gemcitabine for stage IIA pancreatic cancer. The nonspecific diffuse alveolar involvement found on computed tomography (CT), together with the negative tests for infectious aetiology and the continuing severe respiratory failure despite a long course of broad-spectrum therapy, suggested gemcitabine-induced acute pneumonia as the most likely diagnosis.

Thus, after the failure of steroids and all other conventional therapies, the patient was treated with imatinib mesylate on the basis of its activity in the management of graft-versus-host-induced lung fibrosis. A follow-up CT scan of chest one month later showed complete resolution of pneumonia.

**Conclusion:**

Despite the low frequency of serious pulmonary toxicity, gemcitabine widespread use warns clinicians to consider this life-threatening toxicity. The favourable clinical outcome with IM treatment was remarkable, warranting additional study of IM in the treatment of lung fibrosis.

## Background

Pancreatic cancer is one of the most fatal malignancies worldwide, with an annual incidence rate almost identical to the mortality rate; being radical surgical resection the only chance of cure although with high recurrence rates [1].

An improvement in overall survival of patients submitted to radical surgery is provided by adjuvant treatment with gemcitabine or bolus 5-fluorouracil, with no differences in effectiveness between the two, but a better safety profile of gemcitabine [2]. The most common gemcitabine dose-limiting toxicity is myelosuppression, whereas pulmonary toxicity has been reported in less than 10 % of patients [3].

We report the case of a patient who developed an acute gemcitabine induced acute interstitial pneumonia and who was completely solved with imatinib mesylate (IM) after the failure of conventional therapies.

## Case presentation

### Case

A 69-year-old man with a history of 75-pack year cigarette smoking up to 2007 and a consequential centrilobular emphysema presented with jaundice in September 2013. After a thorough staging, he was subjected to pancreaticoduodenectomy. The pathology report showed a mild differentiated pancreatic adenocarcinoma pT3, pN0, stage IIA (TNM 7^th^ Edition).

**Fig. 1:**
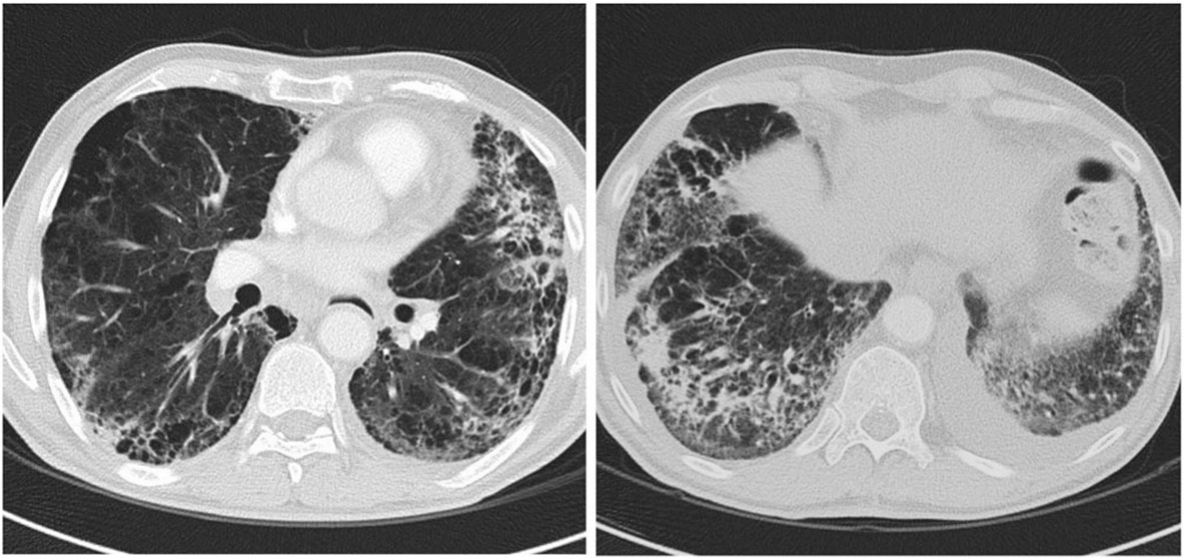
Chest CT scan image obtained at the time of clinical presentation with respiratory symptoms reveals nonspecific diffuse alveolar involvement

On January 2014 we began adjuvant chemotherapy with gemcitabine 1,000 mg/m^2^ on days 1, 8, 15 every 28. The treatment was well tolerated until 1 week after the fourth cycle, when he developed fever up to 38 °C, productive cough and exertional dyspnea. Despite a week of oral levofloxacin (500 mg q24h), dyspnea worsened and he was admitted to our inpatient ward. On examination he was afebrile, with blood pressure of 140/80 mmHg, pulse rate of 90 beats/min and respiratory rate of 22 breaths/min. His oxygen saturation was 85 % on room air, which improved to 94 % after oxygen supplementation via a Venturi mask with a fraction of inspired oxygen of 40 %. On auscultation, he presented diffuse crackles involving lower lung fields. The WBC was 9,350/microL (reference range 4,500 to 10,000/microL), haemoglobin 12.4 g/dl (reference range 13.8 to 17.2 g/dL) and platelet count 360,000/L (reference range 150,000 to 400,000/L). Fibrinogen was 538 mg/dl (reference range < 400 mg/dl), and C-reactive protein level was 0.008 g/L (reference range < 0.005 g/L). The remaining laboratory tests were unremarkable. Room air arterial blood gas analysis revealed oxygen tension of 43 mmHg, carbon dioxide tension of 41 mmHg, bicarbonate 27.7 mmol/L and pH of 7.45. The computed tomography (CT) scan showed several ground-glass opacities, multiple areas of parenchymal consolidation and air bronchogram in lower lobes and; left pleural effusion and multiple enlarged hilar and mediastinal lymph nodes (Fig. 1). Altogether, the CT findings were consistent with nonspecific diffuse alveolar involvement; without signs of pulmonary oedema, embolism, hypertension or tumor lymphangitic spread.

Sputum and blood cultures, CMV-DNA search, streptococcus pneumoniae and legionella urinary antigen and serum aspergillum antigen were negative. An empirical broad-spectrum antimicrobial therapy was started, involving Piperacillin/Tazobactam (4.5 g q6h), Moxifloxacin (400 mg q24h) and Fluconazole (100 mg q24h).

After 7 days, a chest CT showed increasing of parenchymal consolidation and pleural effusion thus pneumologist and infectious disease consultants suggested to change therapy into meropenem (1 g q8h), vancomycin (500 mg q6h), linezolid (600 mg q12h), acyclovir (800 mg q12h), and voriconazole (200 mg q12h).

**Fig. 2:**
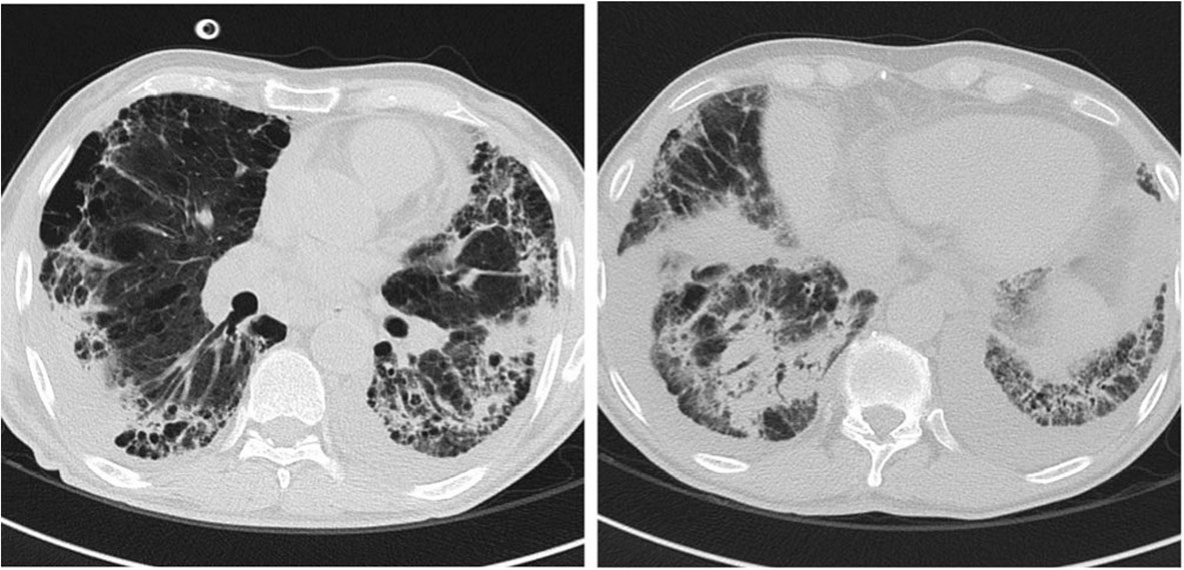
Chest CT scan image after 14 days of broad-spectrum therapy with deteriorating respiratory condition: increase in size and number of the parenchymal consolidation and ground-glass areas, consistent with ongoing pneumonia

In spite of 14 days of broad-spectrum therapy, patient’s respiratory condition continued to deteriorate, a bronchoscopic assessment or lung biopsy was contraindicated, but a further CT scan showed significant increase of parenchymal consolidation and ground-glass areas predominantly in lower lobes, consistent with ongoing pneumonia (Fig. 2).

Considering the CT scan, the negative tests for infectious aetiology and the continuing severe respiratory failure despite a total of 22 days of broad-spectrum therapy, gemcitabine-induced acute pneumonia was considered to be the most likely diagnosis.

Seven days after the start of systemic methylprednisolone 2 mg/kg/die, however, the respiratory condition remained critical, with PaO2 of 65.2 mmHg on arterial blood gases in 60 % mist mask ventilation (15 l/min).

Therefore, on the basis of IM activity in graft-versus-host–induced lung fibrosis treatment, and our previous experience in a similar condition we asked patient consent and started therapy with IM 100 mg daily [4]. After 4 days the clinical condition and respiratory function significantly improved, allowing the reduction of oxygen and steroid therapy until the complete weaning 10 days after, and the discontinuation of IM after 1 month.

**Fig. 3:**
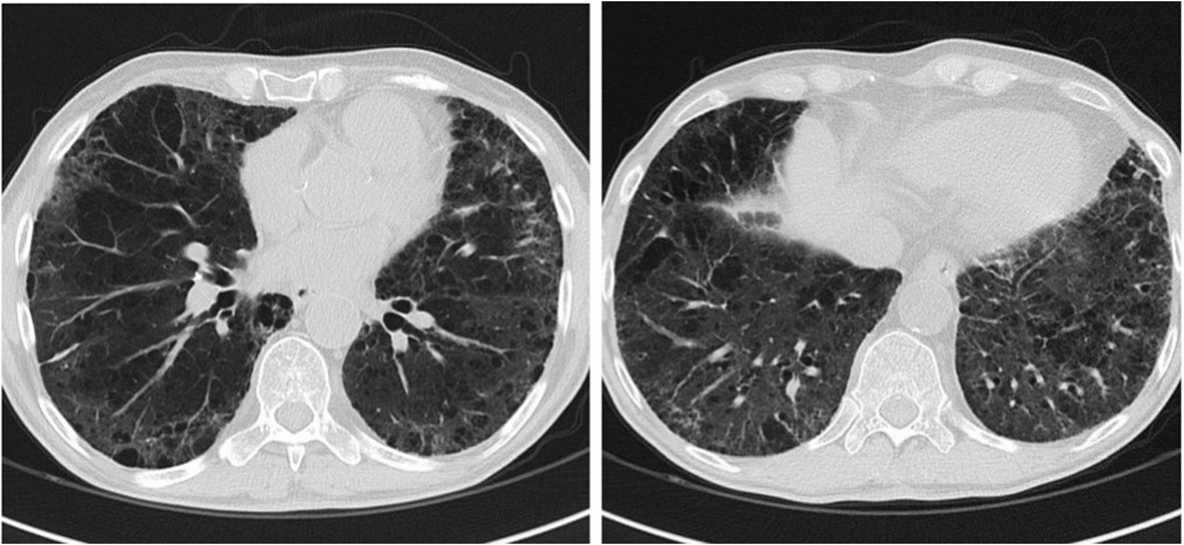
Chest CT scan image obtained 1 month after the start of imatinib mesylate demonstrates resolution of ground-glass opacities and parenchymal consolidation

Due to gemcitabine-induced pulmonary toxicity the adjuvant treatment was discontinued. CT scans after one (Fig. 3) and 5 months showed complete resolution of the alveolar opacities. At the most recent follow-up, the patient is alive without evidence of pancreatic disease, with a Karnofsky performance status of 100 %.

### Discussion

Gemcitabine-induced pulmonary toxicity is a relatively uncommon complication, associated with significant morbidity and mortality [3, 5]. Moreover the incidence appears to be underestimated, since risk factors are unsettled and isolated CT findings inadequate to make the diagnosis [6].

The spectrum of respiratory toxicity varies from mild dyspnea (25 %) to a form of fatal acute respiratory distress syndrome–like picture (0.3 %) [7, 8]. Belknap et al. reviewed data on clinical characteristics of gemcitabine-induced pulmonary toxicity identifying most frequent symptoms in dyspnea (70 %), fever (35 %), and pulmonary infiltrate (21.9 %); with a median time to the diagnosis of 48 days after initiation of gemcitabine [9].

The exact pathogenesis of lung injury is not known, however gemcitabine seems to induce the release of pro-inflammatory cytokines causing deregulation of tissue repair [10, 11]. Specifically, in idiopathic pulmonary fibrosis, an enhanced expression of platelet-derived growth factor receptor (PDGFR) and tumor growth factor (TGF)-beta induces proliferation and transformation of extracellular matrix in idiopathic pulmonary fibrosis [12]. This phenomenon leads to the replacement of normal lung by cystic spaces separated by thick fibrous tissue until progressive cleavage of alveolar structures. In animal models, the same receptors are involved in bleomicine-induced pulmonary fibrosis, which is a pathologic condition that closely resembles the final lung damage observed in gemcitabine-induced pulmonary toxicity.

The management of patients affected by idiopathic pulmonary fibrosis is notably challenging, due to the lacking of a current recommended treatment. Multiple drugs have been tested for pulmonary fibrosis treatment and evidence both for and against their use has alterned over the years. Yet, until recently, this was essentially an untreatable disease. According to existing guidelines, at present there are essentially two therapies that have shown some efficacy. Pirfenidone, an orally administered pyridone compound with a supposable effect on TGF-β production, reduced disease progression and increased survival in this group of patients. Similarly, Nintedanib, a multikinase inhibitor, decelerated disease progression in a comparable cluster. This is a notably breakthrough for patients and physicians alike; nevertheless, the results in these trials were based on clinical outcomes, without a definitively demonstration of per se reduction in lung fibrosis [13].

IM is a specific receptor tyrosine-kinase-inhibitor of both isoforms of PDGFR, approved for chronic myelogenous leukaemia and gastrointestinal stromal tumours. The recognized role of PDGFR and TGF-β pathways in idiopathic pulmonary fibrosis pathogenesis led to subsequent investigations assessing IM as a potential inhibitor of lung fibrosis [14]. Hence IM was identified as a potent inhibitor of lung fibroblast–myofibroblast and extracellular matrix proliferation, showing to prevent bleomycin-induced pulmonary fibrosis in mice.

The hypothesis of treating lung fibrosis using IM has been evaluated in a phase II randomized placebo-controlled trial. Notwithstanding, patients with idiopathic pulmonary fibrosis treated with IM 600 mg once daily, despite a significant increase of PaO2, did not receive significant survival improvement [15]. However it should be emphasized the profoundly different context from our case: patients with idiopathic fibrosis included in that trial had a mild-moderate fibrosis, diagnosed likewise 36 months before, with PaO2-values above 60 mmHg.

These assumptions encouraged us to test a low-toxicity profile drug such as IM in a patient whose rapidly worsening clinical condition had been refractory to previously therapies.

## Conclusions

As discussed above, gemcitabine-induced pulmonary toxicity is a diagnosis of exclusion and several other respiratory diseases have to be ruled out. Nevertheless, this condition needs to be promptly recognized because it may be fatal [16]. Therefore, despite the infrequency of this complication, gemcitabine widespread use warns clinicians to consider this life-threatening toxicity.

The description of a few cases of favourable clinical outcome in literature, including the complete response of our case report, suggests that additional study of IM in the treatment of lung fibrosis are needed, also considering the mentioned lack of efficient treatments.

It is clear that, even though only a randomised-controlled-trial can provide a formal and statistically valid demonstration of the role of IM in this circumstance, such a study is difficult to conceive in a rapidly fatal disease.

## Abbreviations

CMV: Cytomegalovirus
CT: Computed tomography
IM: Imatinib mesylate
PDGFR: Platelet-derived growth factor receptor
TGF-beta: Tumor growth factor
TNM: Tumor nodes metastases
